# A Simple Model to Relate the Elastic Ratio Gamma of a Critically Self-Organized Spring-Block Model with the Age of a Lithospheric Downgoing Plate in a Subduction Zone

**DOI:** 10.3390/e22080868

**Published:** 2020-08-07

**Authors:** Jennifer Perez-Oregon, Alejandro Muñoz-Diosdado, Adolfo Helmut Rudolf-Navarro, Fernando Angulo-Brown

**Affiliations:** 1 Departamento de Física, Escuela Superior de Física y Matemáticas, Instituto Politécnico Nacional, UP Zacatenco, Mexico City 07738, Mexico; ahrudolf@gmail.com (A.H.R.-N.); angulo@esfm.ipn.mx (F.A.-B.); 2 Solid Earth Physics Institute, Physics Department, National and Kapodistrian University of Athens, Panepistimiopolis, Zografos, 157 84 Athens, Greece; 3 Unidad Profesional Interdisciplinaria de Biotecnología, Instituto Politécnico Nacional, Mexico City 07340, Mexico

**Keywords:** self-organized criticality, Ruff–Kanamori diagram, spring-block model

## Abstract

In 1980, Ruff and Kanamori (RK) published an article on seismicity and the subduction zones where they reported that the largest characteristic earthquake ($M_{w}$) of a subduction zone is correlated with two geophysical quantities: the rate of convergence between the oceanic and continental plates (*V*) and the age of the corresponding subducting oceanic lithosphere (*T*). This proposal was synthetized by using an empirical graph (RK-diagram) that includes the variables $M_{w}$, *V* and *T*. We have recently published an article that reports that there are some common characteristics between real seismicity, sandpaper experiments and a critically self-organized spring-block model. In that paper, among several results we qualitatively recovered a RK-diagram type constructed with equivalent synthetic quantities corresponding to $M_{w}$, *V* and *T*. In the present paper, we improve that synthetic RK-diagram by means of a simple model relating the elastic ratio *γ* of a critically self-organized spring-block model with the age of a lithospheric downgoing plate. In addition, we extend the RK-diagram by including some large subduction earthquakes occurred after 1980. Similar behavior to the former RK-diagram is observed and its SOC synthetic counterpart is obtained.

## 1. Introduction

**Figure 1:**
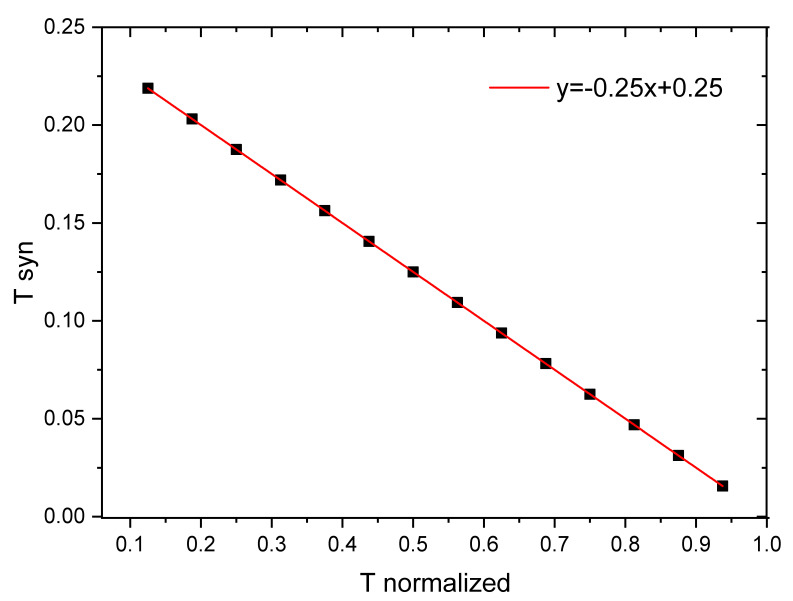
Linear fit between synthetic age ($T_{syn}\equiv \gamma )$ and normalized age (T normalized).

As Kanamori asserts [1], subduction zones with great earthquakes are strongly coupled, and conversely, those without strong earthquakes are weakly coupled. As representative cases of both categories, Uyeda and Kanamori [2] called them of the Chilean-type and of the Marianas-type, respectively (see Figure 2 of [1]). In 1980, Ruff and Kanamori (RK) [3] published an article on seismicity and the subduction zones where they reported that the largest characteristic earthquake ($M_{w}$) of a subduction zone is correlated with two geophysical quantities: the rate of convergence between the subducting oceanic and the overriding continental plates (*V*) and the age of the corresponding subducting oceanic lithosphere (*T*). In Figure 1 of RK [3] the location of the characteristic largest earthquakes for 21 subduction zones around the world are shown and in their table 1, their corresponding $M_{w}$, depth, rupture length, age of the subducting plate and the convergence rate are listed. First, RK tried correlating $M_{w}$ with the depth and lateral extent of the subducting slab, the age of the subducting slab, and the convergence velocity. Individually these correlations were poor [4]. The only acceptable one was with the two independent variables previously mentioned, *T* and *V*. This relation proposed by RK is:

(1) $$M_{w}^{\prime }=-0.00953T+0.143V+8.01,$$

with *T* in Myr and *V* in cm/year. We took the original RK data and could not reproduce it exactly; however the one we got is very similar: $M_{w}^{\prime }=-0.008786T+0.1339V+7.946$, with $R^{2}$ = 0.6427 and the standard deviation std=0.4494.

**Figure 2:**
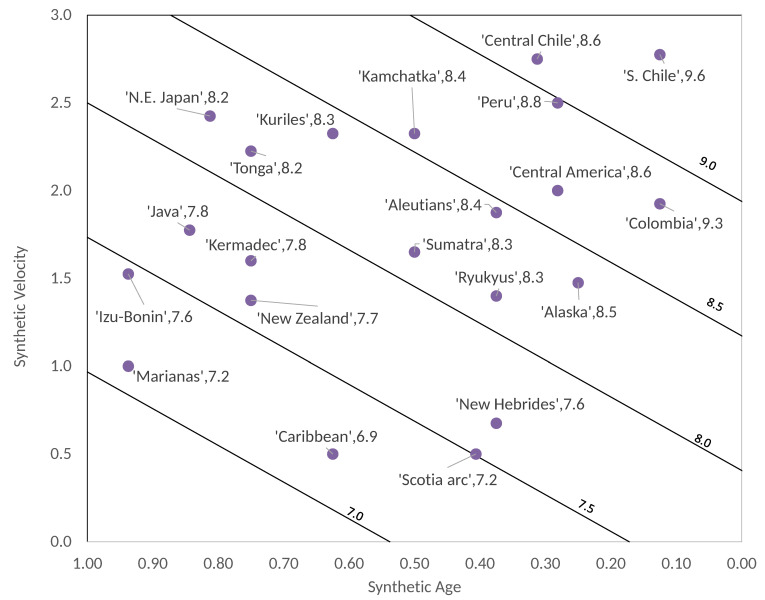
Synthetic Ruff–Kanamori diagram by using Equation (7).

Figure 4 of Kanamori [1] shows the comparison of observed $M_{w}$ with those calculated by Equation (1). As Kanamori’s asserts [1], it should be noted that Equation (1) is obtained empirically without any particular physical model. Several authors [5,6,7,8,9,10,11,12,13,14], have shown that, by means of 2-D spring-block self-organized critical numerical models, it is possible to obtain well-behaved Gutenberg–Richter (GR) type relationships [15], this being a good indication that the Earth’s crust behaves like a self-organized critical (SOC) system. In fact, Geller et al. [16] recognized that there is consensus that this is the case of the Earth’s crust. We have recently published an article [13] in which we go one step further on the possible agreement between a spring-block SOC model and some properties of seismicity in subduction zones. In that article, by using the Olami, Feder and Christensen (OFC) SOC model [10] we were able to reproduce the Ruff–Kanamori diagram that links $M_{w}$ with *T* and *V* (see Figure 2 of [3]). However, in 2011 Heuret et al. [17] stated that a bilinear correlation like the one given by Equation (1) does not hold based on global earthquake catalogues for a period between 1900 and 2007. However, in Figure 10 of [17] present an equivalent RK-diagram where there is apparently no significant correlation between $M_{w}$ and the plate age *T*, but if one calculates a bilinear fit between $M_{w}$, *T* and *V* different scenarios can be obtained, depending on the values taken for $M_{w}$, in some of those scenarios there are significant correlations and in other scenarios the correlation is not significant. In this diagram, Heuret et al. plotted 54 points for *T* and *V* and the values of $M_{w}$ are each specified in an interval, for example, if the values are taken approximately in the middle of the intervals, one of the obtained equations is:

(2) $$M_{w}=-0.002849T+0.0605V+7.887,$$

with $R^{2}$ = 0.5689, and std=0.2177, and when we test for the significance of multiple regression [18] we obtain significant correlation. As in Equation (1) the coefficient of *T* is negative and the coefficient of *V* is positive. As Scholz [4] says, on balance it appears that seismic coupling correlates positively with the convergence rate and negatively with the slab age, only taken together. As we said above, we have recently published an article in which we provide some possible additional evidence about the self-organized critical nature of actual seismicity [13]. As it is well known, a number of SOC models of the spring-block type reproduce well-behaved Gutenberg–Richter relations analogue to those of actual seismicity [5,6,7,8,9,10,11,12,13,14]. The additional evidence we proposed as a SOC attribute of actual seismicity [13] is the reproduction of a Ruff–Kanamori diagram which describes the correlation between the magnitude ($M_{w}$) of the largest characteristic earthquake of a subduction zone with both the age of the lithospheric downgoing plate and the plate convergence rate [3]. In that paper, we suggested that actual seismicity, stick–slip experiments with sandpapers and a self-organized critical model [10] have in common some dynamical features. To emulate the convergence rate between the parallel planes involved in the OFC SOC model, we add to such a model a new rule to those used by the original authors (see rule 4’ in [13]) and for emulating the age of the tectonic plates and also the weathering of sandpapers we use the elastic ratio *γ* of the OFC SOC model (see Section 2). In this way, we construct an analogue of the Ruff–Kanamori diagram (see Figure 2 of [3] and Figures 2 and 3 of [13]). When a linear multivariate fit was made to the data of the original Ruff–Kanamori diagram the following relationship is obtained: ${M_{w}}^{\prime }=-0.00953T+0.143V+8.01$; that is, a negative correlation between $M_{w}$ and the age T and a positive correlation between $M_{w}$ and the convergence rate V, and the standard deviation (std) between actual $M_{w}$ and predicted ${M_{w}}^{\prime }$ given by the mentioned relationship is std=0.423. When the linear multivariate fit is made to the synthetic data of Figure 3 of [13] the obtained relation is ${M_{syn}}^{\prime }=-4.32623T_{syn}+0.957V_{syn}+6.78$. For this case the standard deviation between $M_{syn}$ and ${M_{syn}}^{\prime }$ results 0.824. That is, approximately twice the value of the standard deviation corresponding to the actual Ruff–Kanamori diagram [3]. In appendix 1 of our paper [13] we proposed a quantitative way to relate the age of the tectonic plates with the elastic ratio *γ* of the OFC SOC model. However, in table 1 of [13] we used only qualitative practical arguments to establish such a relation and the model of appendix 1 of that paper, was only mentioned as a qualitative support to the table 1 of the respective paper. Very recently we have used the model of appendix 1 to recalculate the relationship between the elastic ratio *γ* and the age of tectonic plates involved in the actual Ruff–Kanamori diagram [3]. As a result of this recalculation, we have improved the standard deviation between $M_{syn}$ and ${M_{syn}}^{\prime }$, which is now 0.210; (around a half of the std of actual RK-diagram). In this new calculation, we also change the base of the logarithm used to estimate the magnitude of synthetic earthquakes. In the previous paper [13] we used the base e logarithm, and now we use the base 3 logarithm. The present paper is structured in the following way: in Section 2 we develop the ideas contained in appendix 1 of [13]; in Section 3 we recalculate the Ruff–Kanamori diagram and the multivariate fit is obtained on the light of the new data for $M_{syn}$ and ${M_{syn}}^{\prime }$; in Section 4 we also present an extended version of the RK-diagram that includes some data from the 21st century, with its corresponding synthetic version. Finally, we discuss our results and present some concluding remarks.

## 2. A Simple Model to Relate the OFC Elastic Ratio *γ* with the Age of the Lithospheric Downgoing Plate

In 1992, Olami, Feder and Christensen applied SOC concepts to a spring-block system which emulates the earthquakes generated by the interaction between two tectonic plates [10]. In the OFC model the tectonic plates are taken as two parallel planes with a small relative velocity between them, that is, the OFC model is a dynamic two-dimensional system consisting of blocks connected by springs of elastic constants *K_1_* and *K_2_* in the horizontal plane. The blocks that are not in the border of the grid have a total of four neighbors. Each block is also connected by a spring of elastic constant *K_L_* to a rigid upper plate that moves with a very small constant speed (see Figure 1 of [9] and Figure 1 of [10]). A block will move only when the force on it is greater than some threshold value $F_{th}$ (maximum static friction). The forces are distributed in their four nearby neighbors due to the movement of the block, which can cause a chain reaction composed of more sliding events. The dynamics of the OFC model can be described not by solving the system of coupled differential equations that represents it but by mapping such a system into a cellular automaton. Such automaton consists of a network represented by a grid of size L×L, where in each block a force $F_{i,j}$ acts, with i and j integer numbers between 1 and L. The overall force exerted by the springs on a block (i,j) was proposed by Olami et al. [10] and Christensen and Olami [19] as follows:

(3) $$F_{ij}=K_{1}\left[2x_{i,j}-x_{i-1,j}-x_{i+1,j}\right]+K_{2}\left[2x_{i,j}-x_{i,j-1}-x_{i,j+1}\right]+K_{L}x_{i,j},$$

where $K_{1},\;K_{2}$ and $K_{L}$ are the Hooke stiffness constants. After a local slip at the position (i,j), the force redistribution is expressed by:

(4) $$F_{i\pm 1,j}\;\to {\;F}_{i\pm 1,j}+{\;\delta F}_{i\pm 1,j},\;F_{i,j\pm 1}\;\to {\;F}_{i,j\pm 1}+{\;\delta F}_{i,j\pm 1},\;F_{i,j}\to 0,$$

where δF in the closest neighbors is:

(5) $${\delta F}_{i\pm 1,j}=\frac{K_{1}}{2K_{1}+2K_{2}+K_{L}}F_{i,j}=\gamma_{1}F_{i,j};\;{\delta F}_{i,j\pm 1}=\frac{K_{2}}{2K_{1}+2K_{2}+K_{L}}F_{i,j}=\gamma_{2}F_{i,j};$$

with $\gamma_{1}$ and $\gamma_{2}$ being the elastic ratios. This model reproduces, qualitatively, in the isotropic case ($\gamma_{1}=\gamma_{2}=\gamma$), the Gutenberg–Richter law with an exponent *b* close to 1 for γ=0.20 [10,12]. It has already been stated in [13] that, by using some practical arguments a linear relationship between the *γ* elastic ratio and the age of the tectonic plates can be proposed. However, it can be made evident through a type of demonstration that this linear behavior is possible from the expressions for $\gamma_{1}$ and $\gamma_{2}$ in Equation (5); or $\gamma_{1,2}=\frac{K_{1,2}}{2K_{1}+2K_{2}+K_{L}}$. As is well known, the isotropic case $K_{1}=K_{2}=K_{L}$ implies that γ=0.20. Ruff and Kanamori [3] described the different types of coupling between the descending and upper plates, which can be strong (Chile type) or weak (Marianas type), although coupling can also have intermediate strength between these extremes in other cases. Thus, we can take cases with different values of K. For instance, if in the SOC OFC model we put $K_{1}=K_{2}=K\neq K_{L}$, it leads to $\gamma =\frac{K}{4K+K_{L}}$ or $\gamma =\frac{1}{4+\frac{K_{L}}{K}}$ = $\frac{1}{4\left(1+\frac{K_{L}}{4K}\right)}$. Putting $x=\frac{K_{L}}{4K}$ it can be seen that the assumption $x^{2}<1$ is acceptable since all the K’s are of the same order of magnitude, so, in the case of $K_{L}>K$ most likely $K_{L}<4K$ and then $x^{2}<1$. Therefore, the equation $\gamma =\frac{0.25}{1+x}$ can be approximated by means of the Newton’s binomial approximation, then:

(6) $$\gamma =0.25{\left(1+x\right)}^{-1}\approx 0.25\left(1-x\right).$$

This last equation represents a straight line with a negative slope in the plane x−γ. γ is in the interval [0, 0.25] and x belongs to the interval [0, 1], x is a dimensionless quantity. As we have some evidence (obtained from the results of sandpaper experiments [13,20]), on the relationship of γ with the age of the weathered surfaces, another dimensionless quantity is also proposed in the interval [0, 1] (it is clear that the value 1 corresponds to 160 Myr, the largest age included in the actual RK-diagram) which is defined as the normalized age ($e_{n}$) of the tectonic plates and in this case we take $e_{n}=x$; that is, the last equation is now:

(7) $$\gamma \approx 0.25\left(1-e_{n}\right).$$

In the Ruff–Kanamori chart, tectonic ages are in the range of 0 to 160 Myr. Therefore, by dividing the tectonic age of some plate by the maximum value of the considered time scale, $e_{n}$ is obtained. Therefore, we take Equations (6) and (7) as equivalent.

In our previous paper [13] to reproduce the RK diagram we used a linear relationship between the age of the downgoing plate and the *γ*-values inspired by Figure 6 of that a paper (and Figure 2b of [10]). The “practical” criterion we used for elaborating its corresponding table 1 was the following one: as we can observe in Figure 6 of [13], for the *γ*-elastic ratio in the interval [0.07, 0.20] the linear adjustment with respect to the G-R slope *b* is much better than outside this interval. The step between the values of *γ* in this graph is of 0.01 corresponding to fourteen subintervals between [0.07, 0.20]. On the other hand, in the original Ruff–Kanamori diagram [3], the time scale ranges from [0, 160] Myr spaced every 20 Myr. Then, mainly for practical reasons, we map the 14 *γ*-subintervals to 14 age-subintervals that result in the aforementioned table 1 of [13]. All these arguments are qualitatively derived from the results of sandpaper frictional experiments [20]. However, when we compare that table 1 with the results obtained from Equation (7) the coincidence is only qualitative. For this reason, we decide to recalculate the relation between tectonic ages *T* with the *γ*-values calculated by means of Equation (7). The results are shown in Figure 1 and Table 1 of the present paper.

In the case of Table 1 of our previous paper the linear regression between normalized age and *γ* elastic ratio gives, $\gamma =0.16\left(1.375-e_{n}\right)$, being qualitatively similar to Equation (7) which, however, has better support. In the following section we shall use Equation (7) to recalculate the synthetic RK diagram and the multivariate fit of the variables involved in the RK diagram.

## 3. A Simple Model to Relate the OFC Elastic Ratio *γ* with the Age of the Lithospheric Downgoing Plate

If we use Equation (7) to recalculate the *γ*-values by means of the normalized tectonic ages, we obtain now the values shown in Table 1. If in addition we use the same values shown in table 2 of [13]; that is, the actual convergence rates (left column) and the force increments larger than $F_{th}-F_{max}$ made as a global perturbation, i.e., the ΔF-values (right column) emulating the convergence rates in the OFC SOC model, then we can recalculate the synthetic RK-diagram and also the synthetic multivariate linear fit of the obtained data. As we said in [13], to emulate an increase in the relative velocity between the two plates we add $F_{th}-F_{max}$ as a global perturbation just for once, this proposal makes sense because Pardo and Suárez [21] reported that the relative velocity between the Cocos and the North American plates along of the Mexican Pacific trench increases from the Colima–Jalisco zone until the Chiapas zone, and it is well known that the number of seisms of any magnitude per year along this same section of the Mexican trench has also a crescent behavior. Therefore, the OFC proposal that consists in the way to increase the total force in each block as proportional to the product $K_{L}V$, is reasonable; that is, the greater the relative velocity *V* the greater $F_{i,j}$ on each block and, therefore, the probability that one or many blocks reach or exceed $F_{th}$ increases.

Here, we proceed in the same way as we did in our previous paper. First, we gather in three columns the values of $M_{w},\;T$ and V extracted from the actual RK-diagram (see table 1 of [3]). Then, we map the actual values of these quantities to synthetic ones following a simple procedure which we explain with the example of the tectonic zone called S. Chile. This zone has in the RK-diagram the values $M_{w}=9.5,\;T=20\;\mathrm{Myr}$ and V=11.1 cm/year. The first step is to map the value T=20 Myr ($e_{n}=\frac{20}{160}=0.125)$ in a *γ*-value given by Equation (7), which leads to γ=0.22 and then converting by means of table 2 of [13] the value of V=11.1 cm/year in ΔF=2.78. With these two synthetic values we construct a catalog of $10^{6}$ synthetic earthquakes, then we choose the event with the largest number of perturbed blocks n, which in this case is n=36,595. Next we calculate a synthetic magnitude $M_{syn}$ by selecting an adequate logarithmic base. If we take a base 3 logarithm we have $M_{syn}=log_{3}36595=9.6$; that is, a value very close to $M_{w}=9.5$. The same was made for the rest of largest earthquakes shown in the actual RK-diagram resulting in a synthetic RK-diagram as that depicted in Figure 2.

**Figure 3:**
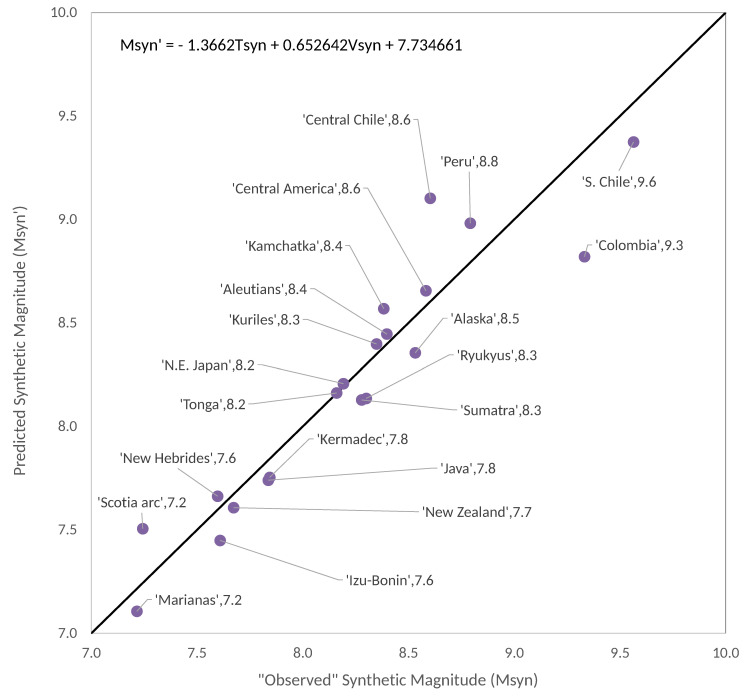
Multivariate linear fit for synthetic seismic zones, for the recalculated data of the Ruff–Kanamori diagram.

As we can see in Figure 2, this synthetic diagram at first glance resembles more the actual RK-diagram than Figure 3 of [13]. On the other side, we can also recalculate the multivariate linear fit given by Figure 5 of [13]. In this case the resulting regression (see Figure 3) is:

(8) $${M_{syn}}^{\prime }=-1.366T_{syn}+0.6526V_{syn}+7.735,$$

with $R^{2}$ = 0.8933, and std=0.2292.

As we said in Section 1, with the approach based in Equation (7) the synthetic results are remarkably improved. The multivariate linear fit obtained by Ruff and Kanamori [3] had a standard deviation of 0.423 between $M_{w}$ and ${M_{w}}^{\prime }$ and the multivariate linear fit between $M_{syn}$ and ${M_{syn}}^{\prime }$ reported in Perez-Oregon et al. [13] had a standard deviation of 0.825. Our new synthetic results shown in Figure 3 have a standard deviation between $M_{syn}$ and ${M_{syn}}^{\prime }$ of 0.2292; that is, a value around half of that obtained by means of the Ruff and Kanamori [3] data. Interestingly, in our Figure 2 we obtained a better resemblance of the actual RK-diagram in the sense that the five diagonal bands contain practically the same largest earthquakes than actual RK-diagram.

## 4. Extended Ruff–Kanamori Diagram

### 4.1. Real Seismicity

Obtaining an updated Ruff–Kanamori diagram would imply (1) obtaining more current values of the subduction velocity, the magnitude of the characteristic maximum earthquake and the age of the corresponding plate. Furthermore, (2) it would be convenient to have data from other seismic regions. Searching the specialized references it is easy to find the $M_{w}$ values of the characteristic largest earthquake; the measurement of the magnitude of a large earthquake is currently performed with great precision. Many of the values in the original Ruff–Kanamori diagram must be updated for the magnitude. Values of *V* are more difficult to obtain, and their values are measured indirectly with higher uncertainties, in fact, in many references the rate is given in an interval. However, the largest uncertainties are in estimating the age of the plates, in most references the ages are given in very wide intervals, often differing from each other by several tens of Myr.

**Figure 4:**
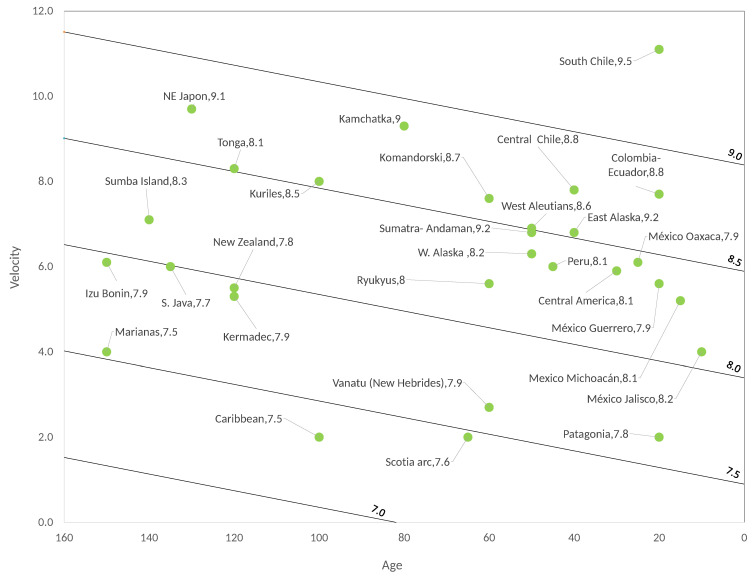
Extended Ruff–Kanamori diagram for real seismicity.

To obtain updated data to construct a new Ruff–Kanamori diagram, $M_{w}$ values were obtained from the USGS website [22]. However, what value should be assigned for the velocity *V* or for the age of the plates when the reported value is in an interval? The average of the maximum and minimum values of those subduction velocity values that are specified as an interval was taken from [23,24,25,26,27,28,29,30,31,32,33,34,35,36,37,38,39,40,41,42,43,44]. The situation is more complicated for *T*, because, what value to assign when the references say T>70 Myr? We used the following rule. As we have published [13] in the synthetic seismicity analysis, there is a bilinear synthetic relationship between $M_{w}$, *T* and *V* and the Ruff–Kanamori diagram can be approximately reproduced using synthetic seismicity. From this relationship, one of the variables can be calculated if the values of the other two are available. However, it would not be appropriate to use this equation to calculate the velocity nor to calculate the magnitude because, as it has been said, the values of age have very large uncertainties associated and the values of the velocity of subduction also have important uncertainties. Instead, calculating the variable *T* from the variables that are best measured can give adequate values for it. Thus, $T_{syn}$ was calculated using the equation obtained from the adjustment of the synthetic seismicity, then the equivalent for real seismicity was obtained and at the end the value that is closest to the obtained value was chosen from the reported interval. It is necessary to make the following assumptions in this methodology: The obtained values for $T_{syn}$ must be between 0 and 0.25 because, in the model, the values of $T_{syn}$ coincide with the values of *γ*. Once the values of $T_{syn}$ have been calculated, the real values of *T* are calculated from Equation (7) as: $T=\frac{0.25-T_{syn}}{0.0016}$, this is because the adjustment of $T_{syn}$ against the real age *T* gives, $T_{syn}=-0.0016T+0.25$. In case we obtained a value of $T_{syn}$ out of the interval [0, 0.25], that is, a negative value or a value greater than 0.25, this is probably due to the fact that the values of *V* are not the most adequate and, in such a case, we take a *V*-value in the interval reported in the references that results in a value of $T_{syn}$ within the interval [0, 0.25]. Fortunately, only two cases with these characteristics were obtained, the Marianas case and the Mexico-Jalisco case. With these considerations, Table 2 is obtained (see also Figure 4).

**Figure 5:**
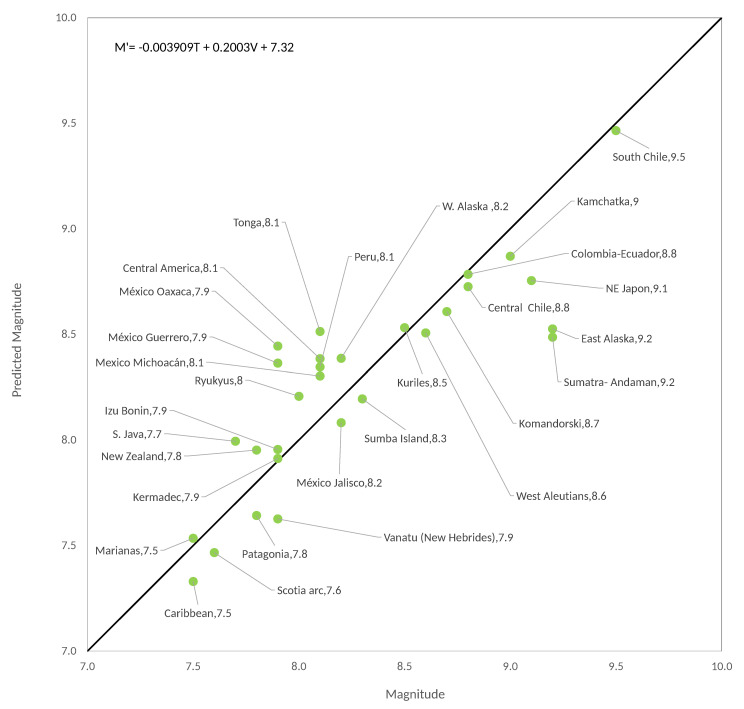
Multivariate linear fit for real seismic zones for the extended data of Ruff–Kanamori.

When making the bilinear adjustment it was obtained (see Figure 5):

(9) $${M_{w}}^{\prime }=-0.003909T+0.2003V+7.32,$$

with $R^{2}=0.7283$ and std=0.3008, which is a value not so distant to that obtained for the original Ruff–Kanamori data [3] (see Section 5), the correlation is significant, although a correlation as high as in the synthetic case is not obtained. The last column of Table 2 shows the calculation of ${M_{w}}^{\prime }$ by using Equation (9); in general, the values are acceptable.

Figure 4 and Figure 5 show the new Ruff–Kanamori diagram and the bilinear fit for the new data, respectively. As in the original adjustment for RK, the correlation between *M_w_* and *T* is negative and between *M_w_* and *V* the correlation is positive, what it simply says is that in the youngest seismic faults earthquakes of great magnitude can occur, while in the old seismic faults earthquakes with smaller magnitudes occur. Similarly, seismogenic zones where the subduction velocity is highest can produce the largest earthquakes such that a combination of young faults (small *T*) with large subduction velocities results in the zones with the highest seismic risk on the planet. It is not possible to be totally conclusive with these statements because we do not have enough data to increase the reliability of the performed statistical calculations, but making the hypothesis test for the multiple regression we find that the correlation is significant with a significance level of 0.05. The other argument in favour of the existence of this bilinear relation between *M_w_*, *T* and *V* has been given using the OFC model in which this bilinear relation has been shown to exist, shown in the following subsection that, for the synthetic version of the extended RK diagram, we also obtained the bilinear relation. This relationship is a consequence of the SOC behavior of the OFC model and the evidence that the Earth’s crust also approaches SOC dynamics that continue to accumulate.

### 4.2. Synthetic Seismicity

**Figure 6:**
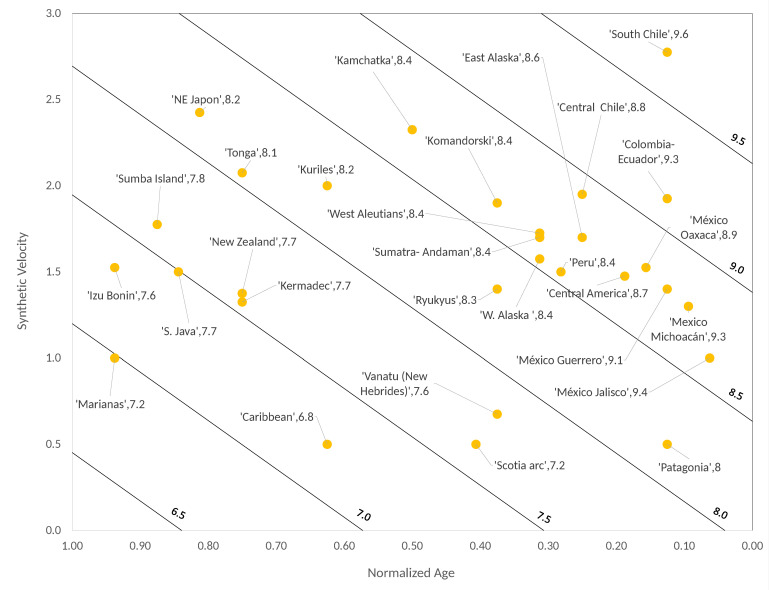
Extended synthetic Ruff–Kanamori diagram.

**Figure 7:**
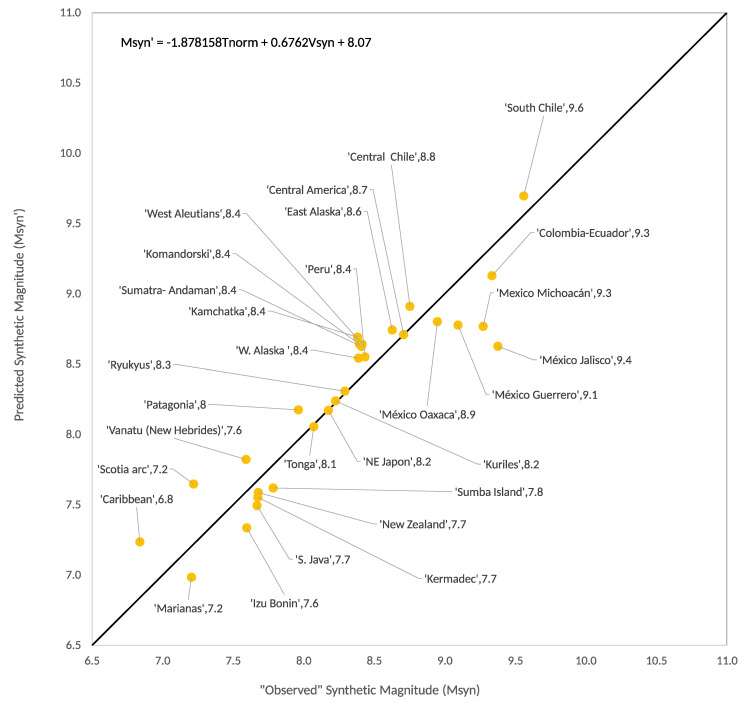
Multivariate linear fit for synthetic seismic zones, for the updated data of Ruff–Kanamori.

Now using the same procedure, described in Section 2 and Section 3, to construct the SOC-OFC synthetic earthquakes of the extended RK diagram we obtain the data shown in Table 3, which give rise to Figure 6 and Figure 7. Figure 6 shows the diagonal bands in the synthetic velocity versus synthetic age plane and Figure 7 shows the bilinear fit between the observed magnitudes ($M_{syn}=log_{3}n$) and the ones calculated by means of the expression:

(10) $${M_{w}}^{\prime }=-1.871T+0.6762V+8.068,$$

with $R^{2}=0.8539$ and std=0.2752.

## 5. Discussion

As Watkins et al. [11] stated, one of the most successful applications of the SOC concept has been in the field of seismicity. In fact, Geller et al. [16] have recognized that in several aspects the Earth’s crust behaves as a critically self-organized system. An indication of this is the way in which some models of the SOC spring-block type reasonably reproduce the Gutenberg–Richter law [5,6,7,8,9,10,11,12,13,14]. This success of the OFC model is strengthened by the following remarks made in [45]: This model is probably the most studied non-conservative, supposedly SOC model, which ironically has been used as an argument that it is not possible to predict the occurrence of large avalanches based on the claim that avalanches seem to be uncorrelated in the original sandpile model. In other words, a belief was expressed that power-law distributed avalanches are inherently unpredictable, which came from the concept of SOC, but interpreted in the way that, at any moment, any small avalanche can eventually cascade to a large event [16]. However, careful and numerical studies [46,47] showed that particularly large events in a close to SOC system can be predicted on the basis of past observations. In addition, it has been shown [48,49] that upon analyzing the OFC model in a new analysis of complex time series, termed natural time analysis—which has been of usefulness to unveil hidden properties in seismicity time series (e.g., [50]) as well as in physiological time series [51]—showed the following very important property: a non-zero change ΔS of the entropy in natural time upon time reversal is identified which reveals a breaking of the time symmetry, thus reflecting the predictability in the OFC model. This has been strikingly confirmed [45] because a non-zero change of ΔS was detected almost two and a half months before the M9 Tohoku mega-earthquake on 11 March 2011 in Japan. 

In addition, recently we have qualitatively reproduced [13] the so-called RK diagram that relates the magnitude $M_{w}$ with the plate age *T* and the convergence rate *V* for a subduction zone by a bilinear equation of the form: $M_{w}=aT+bV+c$, where, both in real seismicity and in synthetic seismicity, it is obtained that a<0 (that is, there is an anti-correlation between $M_{w}$ and *T*) and b>0 (that is, there is a positive correlation between the variables $M_{w}$ and *V*). In fact, the OFC model gives very good results in terms of reproducing Gutenberg–Richter type laws. Another very interesting fact is that this model reproduces RK diagrams with very high $R^{2}$ values and small standard deviations, as seen in Figure 3 and Figure 7. Furthermore, if we include the confidence intervals in the expressions of the bilinear fit inserted in the upper part of these figures, we obtain:

$$a=-1.366\left(-1.794,-0.9387\right)\;b=0.6526\left(0.4907,0.8146\right)\;c=7.735\left(7.324,8.146\right),\;R^{2}=0.8933,\;std=0.2292$$

and:

$$a=-1.871\left(-2.251,-1.492\right)\;b=0.6762\left(0.4815,\;0.8709\right)\;c=8.068\left(7.717,\;8.419\right)\;R^{2}=0.8539,\;std=0.2752$$

As can be seen, the confidence intervals of the corresponding coefficients are not wildly far from each other, despite the fact that they were calculated for a different number of events (21 and 30, respectively). The *R* values for the synthetic seismicity data settings are consistently greater than 0.9, indicating a very good correlation and the standard deviation is small compared to the actual seismic cases.

In regard the bilinear fits of the actual seismicity given by Equations (1), (2) and (9) for the RK, Heuret and the extended RK data, respectively, if we include their confidence intervals we obtain:

$$\mathrm{RK}:\;a=-0.008786\;\left(-0.01402,-0.003546\right)\;b\;=0.1339\;\left(0.05449,\;0.2133\right)\;c=7.946\;\left(7.14,\;8.753\right)\;R^{2}=0.6427,\;std=0.4494\;\mathrm{Heuret}:\;a=-0.002849\;\left(-0.004288,-0.001411\right)\;b\;=0.0605\;\left(0.04404,\;0.07697\right)\;c=7.887\;\left(7.745,\;8.03\right)\;R^{2}=0.5689,\;std=0.2177\;\mathrm{Extended}\;\mathrm{RK}:\;a=-0.003909\;\left(-0.006455,-0.001363\right)\;b\;=0.2003\;\left(0.148,\;0.2526\right)\;c=7.32\;\left(6.944,\;7.696\right)\;R^{2}=0.7283,\;std=0.3008$$

In this case, despite the heterogeneity of the data and their sources, as well as the number of events (21 for RK, 54 for Heuret and 30 for the extended RK), the confidence intervals are not excessively distant from each other. The average of the *R* values is approximately 0.8, in such a way that although the correlations are not as great as in the synthetic cases, in the three cases discussed the correlations are significant. On average, the standard deviations are greater than those obtained in the synthetic case.

From all of the above, we believe that if one has a sufficiently large number of characteristic earthquakes and with sufficiently precise data from convergence rate and age of the tectonic plates will be possible to discern if the seismicity in the subduction zones has an authentic SOC behavior.

## 6. Conclusions

In this paper, we have recalculated a previous version of a synthetic diagram emulating an actual Ruff–Kanamori empirical diagram relating the magnitude $M_{w}$ of the largest characteristic earthquake of a subduction zone with two quantities: the convergence speed between downgoing and upper plates and the age of the downgoing plate. The new calculations are based on a simple model relating the elastic ratio *γ* of a SOC OFC spring-block model with the age of the lithospheric downgoing plate. This model leads to a simple linear relationship between *γ* and the normalized age of the corresponding subducting plate. Once we convert the actual variables $M_{w}$, *T* and V into synthetic equivalent ones, we made a synthetic catalogue formed by one million of events and then we choose the largest event. With the synthetic data we elaborate a RK synthetic diagram obtaining $M_{syn}$ and ${M_{syn}}^{\prime }$ values which have standard deviations very close to those of the actual data $M_{w}$ and ${M_{w}}^{\prime }$.

To reinforce our results we proceeded to build an extended RK diagram, where we included more seismic regions and updated the values $M_{w}$, *V* and *T* with recent references and, following the procedure described in the article, we built a new synthetic version for the RK diagram and in both cases we find evidence of the possible existence of the bilinear relationship of $M_{w}$ with *T* and *V*, and always a negative correlation between $M_{w}$ and *T*, as well as a positive correlation between $M_{w}$ and *V*. There is not enough data to be totally conclusive about the existence of the bilinear relationship, but what we do affirm is that such a relationship cannot be completely ruled out. In the OFC model such a bilinear relationship is observed, this model is critically self-organized and there is evidence that the Earth’s crust is also SOC, which, according to ourselves, makes the existence of such a relationship more plausible, indicating that, on average, the earthquakes with the highest magnitude should occur where *V* is larger and where tectonic plates are younger.

Another advantage of the possible existence of such a bilinear relationship is that it allows the variable *T* to be calculated, which is usually determined with a high degree of uncertainty, in terms of the variables $M_{w}$ and *V* whose determination is made with greater precision.

## Figures and Tables

**Table 1:** The lithospheric age range reported in the RK-diagram [3] is shown in the first column. The normalized age is shown in the second column and in the third column the γ -values of the OFC model that emulates the aging effect are provided. See Equation (7).

Age T (Myr)	T Normalized	$T_{syn}$ (γ-Values)
20	0.125	0.219
30	0.188	0.203
40	0.250	0.188
50	0.313	0.172
60	0.375	0.156
70	0.438	0.141
80	0.500	0.125
90	0.563	0.109
100	0.625	0.094
110	0.688	0.078
120	0.750	0.063
130	0.813	0.047
140	0.875	0.031
150	0.938	0.016

**Table 2:** Extended RK diagram data. The magnitudes of the maximum reported earthquakes were taken from the USGS [22]. Numbers in brackets correspond to the references from where the subduction rate and the age were taken.

Subduction Seismic Region	Subduction Rate V (cm/Year)	Magnitude $M_{w}$ of the Maximum Reported Earthquake	Age (Myr)	Calculated $M_{w}$
East Alaska [23,24]	6.8	9.2	40	8.5
West Aleutians [23]	6.9	8.6	50	8.5
Sumatra-Andaman [25]	6.8	9.2	50	8.5
Central Chile [26,27]	7.8	8.8	40	8.7
South Chile [3,26,27]	11.1	9.5	20	9.5
Marianas [17]	4	7.5	150	7.5
Peru [28]	6.0	8.1	45	8.3
Tonga [17,26,29,30]	8.3	8.1	120	8.5
Colombia-Ecuador [3,31]	7.7	8.8	20	8.8
Central America [21,28,32]	5.9	8.1	30	8.4
NE Japan [3,33]	9.7	9.1	130	8.8
Kamchatka [3]	9.3	9	80	8.9
S. Java [34,35]	6.0	7.7	135	8.0
Izu Bonin [3,36]	6.1	7.9	150	8.0
Kermadec [37]	5.3	7.9	120	7.9
Kuriles [29]	8	8.5	100	8.5
New Zealand [3,38]	5.5	7.8	120	8.0
Vanatu (New Hebrides) [3,39]	2.7	7.9	60	7.6
Ryukyus [40,41]	5.6	8	60	8.2
Caribbean [3,42]	2	7.5	100	7.3
Scotia arc [3,43]	2	7.6	65	7.5
W. Alaska [23,26]	6.3	8.2	50	8.4
Komandorski [23,26]	7.6	8.7	60	8.6
Patagonia [17,43]	2	7.8	20	7.6
México- Jalisco [21,26,28]	4	8.2	10	8.1
Mexico-Michoacán [21,26,28]	5.2	8.1	15	8.3
México- Guerrero [21,26,28]	5.6	7.9	20	8.4
México- Oaxaca [21,26,28]	6.1	7.9	25	8.4
Sumba Island [34,44]	7.1	8.3	140	8.2

**Table 3:** Synthetic extended RK diagram data.

Subduction Seismic Region	*T_syn_* (Gamma)	Normalized Age *e_n_*	*V_syn_*	Maximum Size (*m*) of Synthetic Earthquake	Observed $M_{syn}=log_{3}n$	Predicted *M_syn_*′
East Alaska	0.188	0.25	1.700	13,084	8.6	8.7
West Aleutians	0.172	0.31	1.725	10,352	8.4	8.6
Sumatra-Andaman	0.172	0.31	1.700	10,283	8.4	8.6
Central Chile	0.188	0.25	1.950	15,020	8.8	8.9
South Chile	0.219	0.13	2.775	36,428	9.6	9.7
Marianas	0.016	0.94	1.000	2738	7.2	7.0
Peru	0.180	0.28	1.500	10,572	8.4	8.6
Tonga	0.063	0.75	2.075	7099	8.1	8.1
Colombia-Ecuador	0.219	0.13	1.925	28,450	9.3	9.1
Central America	0.203	0.19	1.475	14,301	8.7	8.7
NE Japan	0.047	0.81	2.425	7959	8.2	8.2
Kamchatka	0.125	0.50	2.325	9994	8.4	8.7
S. Java	0.039	0.84	1.500	4561	7.7	7.5
Izu Bonin	0.016	0.94	1.525	4216	7.6	7.3
Kermadec	0.063	0.75	1.325	4598	7.7	7.6
Kuriles	0.094	0.63	2.000	8405	8.2	8.2
New Zealand	0.063	0.75	1.375	4610	7.7	7.6
Vanatu (New Hebrides)	0.156	0.38	0.675	4187	7.6	7.8
Ryukyus	0.156	0.38	1.400	9043	8.3	8.3
Caribbean	0.094	0.63	0.500	1832	6.8	7.2
Scotia arc	0.148	0.41	0.500	2786	7.2	7.6
W. Alaska	0.172	0.31	1.575	10,054	8.4	8.5
Komandorski	0.156	0.38	1.900	10,124	8.4	8.6
Patagonia	0.219	0.13	0.500	6298	8.0	8.2
Mexico-Jalisco	0.234	0.06	1.000	29,780	9.4	8.6
Mexico-Michoacán	0.227	0.09	1.300	26,583	9.3	8.8
Mexico-Guerrero	0.219	0.13	1.400	21,842	9.1	8.8
Mexico-Oaxaca	0.211	0.16	1.525	18,605	8.9	8.8
Sumba Island	0.031	0.88	1.775	5175	7.8	7.6
